# Ultrasonographic diagnosis and surgical outcomes of adnexal masses in children and adolescents

**DOI:** 10.1038/s41598-022-08015-4

**Published:** 2022-03-10

**Authors:** Gun Gu Kang, Kyeong A So, Ji Young Hwang, Nae Ri Kim, Eun Jung Yang, Seung Hyuk Shim, Sun Joo Lee, Tae Jin Kim

**Affiliations:** Department of Obstetrics and Gynecology, Konkuk University School of Medicine, 120-1, Neungdong-ro, Gwangjin-gu, Seoul, 05030 Republic of Korea

**Keywords:** Medical research, Signs and symptoms

## Abstract

This study aimed to evaluate the incidence, clinical diagnosis, surgical treatment, and histopathological findings of adnexal masses in children and adolescents. This retrospective study included patients aged < 20 years who were diagnosed with adnexal masses between January 2005 and December 2018 at the Konkuk University Medical Center. Adnexal masses were diagnosed in 406 patients. The mean age of patients was 17.3 years at the time of diagnosis. The primary presenting symptoms and signs were abdominal pain (81.4%), mass per abdomen (13.7%), dysmenorrhea (3.4%), incidental finding (2%), and abdominal distention (0.5%). In total, 204 patients underwent surgery for adnexal masses, and 202 patients were observed without surgery. Histopathological examination revealed 110 benign neoplasms, 72 non-neoplastic lesions, 3 ectopic pregnancies, 3 tubo-ovarian abscesses, 7 borderline malignant tumors, and 9 non-epithelial ovarian malignant tumors. Abdominal pain was the most common reason for hospital visits and surgery in adolescents and young women with adnexal masses. The ultrasonographic diagnosis was consistent with the histopathological diagnosis. In recent years, the use of minimally invasive surgery such as laparoscopy and robotic, has increased in young patients with adnexal masses.

## Introduction

Adnexal masses are uncommon in children and adolescents. The annual incidence of adnexal masses is approximately 2.6 cases per 100,000 girls^1^. Adnexal masses may represent physiological ovarian cysts, tubal origin, or neoplasms. In addition, adnexal masses are associated with ectopic pregnancy or pelvic inflammatory diseases. Gynecologists are less familiar with the management of adnexal masses in these age groups compared to that of adults because of its low incidence and varied etiologies^2^.

Most adnexal masses in children and adolescents are non-neoplastic ovarian cysts, including follicular cysts, corpus luteum, and theca lutein cysts, due to irregular menstruation and frequent anovulation. Benign neoplasms are more common than malignant neoplasms. The most frequent types of benign neoplasms include mature teratomas, mucinous and serous cystadenomas, and endometriomas^3^. Malignant ovarian tumors account for only 0.9% of all childhood and adolescent malignancies^4^. Unlike adults, approximately 80% of ovarian malignancies are germ cell tumors, and the remaining tumors are derived from epithelial and sex cord-stromal cells in children and adolescents^5^.

The clinical symptoms and signs vary and are non-specific in young women with adnexal masses. It is necessary to determine the possibility of malignant tumors using multimodal diagnostic methods, including serum tumor markers, ultrasonography, computed tomography (CT), and magnetic resonance imaging (MRI)^5,6^. Occasionally, a surgical approach may be required for the diagnosis of malignancy. Surgical treatment for benign ovarian tumors is minimally invasive, and ovarian-sparing surgery is preferred over oophorectomy because of the fertility preservation benefits in this age group^7^. In contrast, malignant ovarian tumors should be treated with exploratory staging surgery and salpingo-oophorectomy for complete surgical removal of the mass. Standard staging surgery is considered only when there is a high risk of malignancy due to an extremely low incidence of ovarian malignancies in this population.

In this study, we investigated the incidence, clinical features, surgical treatment, and histopathological findings of adnexal masses in children and adolescents over 14 years, with the aim of improving the clinical management of adnexal masses in this population.

## Results

During the study period, 406 children and adolescents were diagnosed with adnexal masses (Table 1). The mean patient age was 17.3 ± 2.7 (range, 5–20) years at the time of diagnosis. The primary presenting symptoms and signs were abdominal pain (87.4%), abdominal mass (6.7%), dysmenorrhea (3.0%), incidental finding (2%), and abdominal distention (0.5%). Adnexal masses were found on the right side in 55.9%, left side in 34.5%, and were bilateral in 9.6% patients. The mean maximum size of the mass was 5.9 ± 4.1 cm. Ultrasonographic findings showed that 253 (62.3%) patients had non-neoplastic tumors, 130 (32.0%) patients had benign tumors, and 23 (5.7%) patients had malignant tumors.

**Table 1:** Patient characteristics (n = 406).

Category	Number
Age (years, range)	17.3 ± 2.7 (5–20)
**Clinical presentation**	
Abdominal pain	355 (87.4%)
Abdominal mass	27 (6.7%)
Dysmenorrhea	12 (3.0%)
Incidental finding	10 (2.5%)
Abdominal distension	2 (0.5%)
**Sonographic findings**	
Tumor size (cm, range)	5.9 ± 4.1 (1.0–3.6)
Site of mass	
Right	227 (55.9%)
Left	140 (34.5%)
Bilateral	39 (9.6%)
Diagnosis	
Non-neoplastic tumor	253 (62.3%)
Benign-neoplastic tumor	130 (32.0%)
Malignant tumor	23 (5.7%)
**Surgery**	
Yes	204 (50.3%)
No	202 (49.8%)

In total, 204 patients underwent surgery for adnexal masses, and 202 patients were observed without surgery. Significant differences between the observation and surgery groups were found in the size of the mass (3.7 ± 1.4 cm vs. 8.1 ± 4.6 cm, *P* < 0.0001) and clinical diagnosis based on ultrasonography (non-neoplastic tumor vs. neoplastic tumor, *P* < 0.0001) (Table 2). No differences were found in age (*P* = 0.589) or symptoms (*P* = 0.542) between the groups. The mean follow-up period was 16.5 months in the observation group. Most patients in the observation group demonstrated spontaneous regression of ovarian cysts, including hemorrhagic cysts, corpus luteum cysts, functional cysts, benign cysts, and tubo-ovarian abscesses. Seven patients with endometriosis and one patient with tubo-ovarian abscess had lesions persistent for > 30 days.

**Table 2:** Clinical differences between observation and surgery groups.

Category	Observation (n = 202)	Surgery (n = 204)	*P*-value
Age	17.4 ± 2.6	17.3 ± 2.8	0.589
Tumor size (cm)	3.7 ± 1.4	8.1 ± 4.6	< 0.0001
**Symptoms**			
No	6 (3.0%)	4 (2.0%)	0.542
Yes	196 (97.0%)	200 (98%)	
**Diagnosis on sonography**			
Non-neoplastic tumor	180 (89.1%)	68 (33.3%)	< 0.0001
Neoplastic tumor	22 (10.9%)	136 (66.7%)	

**Figure 1:**
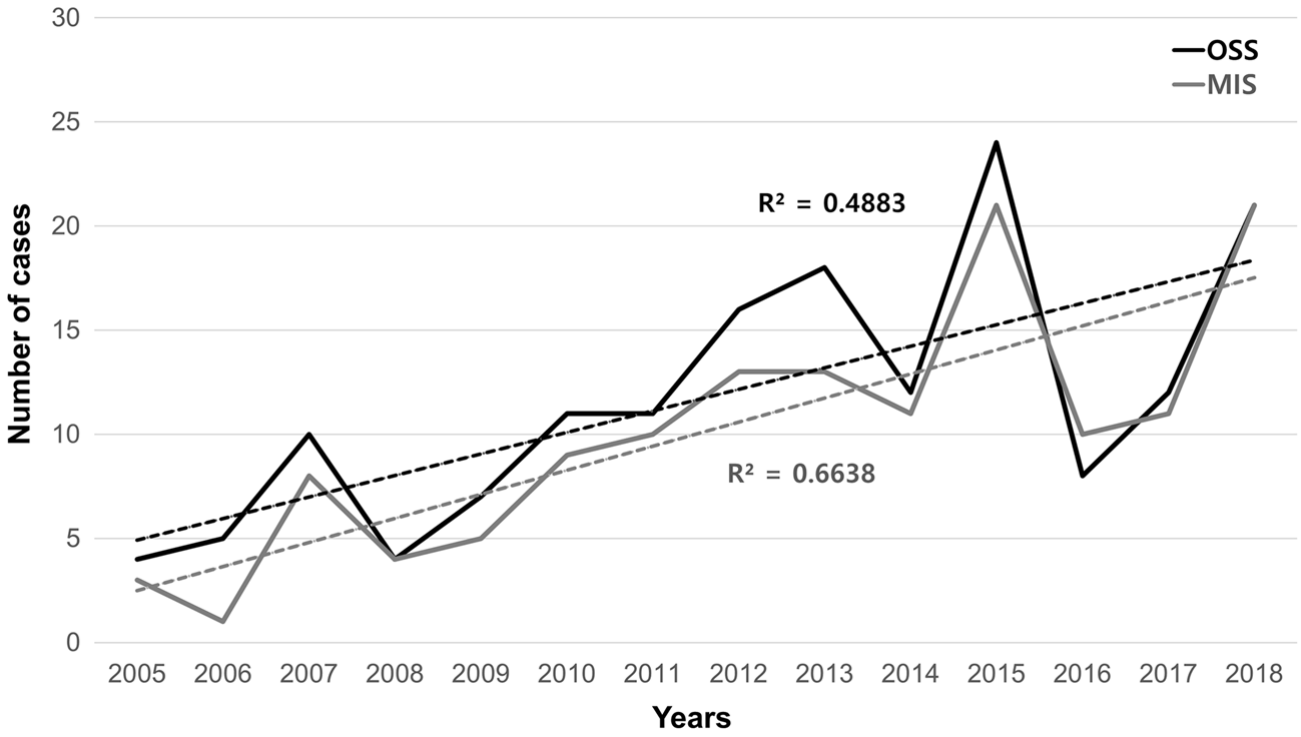
The trends of surgical treatment for adnexal masses in children and adolescents. Ovarian-sparing surgery (OSS) indicated by black dash line. Minimally invasive surgery (MIS) indicated by gray dash line.

The clinical characteristics of the patients undergoing surgical treatment are shown in Table 3. Elective surgery was performed more frequently than emergency surgery (60.8% vs. 39.2%). On histopathology, 72 (35.3%) patients had non-neoplastic tumors, 110 (53.9%) patients had benign neoplasms, 16 (7.8%) patients had malignant tumors, 3 (1.5%) patients had ectopic pregnancies, and 3 (1.5%) patients had tubo-ovarian abscesses. The diagnostic agreement between the initial ultrasonography and histopathology was significant (Cohen's kappa, k = 0.722, *P* < 0.0001). Ultrasound examinations were performed by 14 gynecologist who have undertaken at least 3000 ultrasonography per year during the study period. The agreement between ultrasound findings and pathologic diagnosis was 82.4% (range, 76.5–85.0%). Surgical treatments included cystectomy (79.9%), oophorectomy or salpingo-oophorectomy (11.3%), salpingectomy (7.4%), and cytoreductive surgery (1.5%). Minimally invasive surgery (MIS) (laparoscopy or robotic surgery, 76.0%) was performed more frequently than laparotomy (24.0%). A gradually increasing trend toward ovarian conservation and MIS in children and adolescents was observed from 2005 to 2018 (Fig. 1).

**Table 3:** Clinical characteristics of patients with surgical treatment (n = 204).

Category	Number
Duration from diagnosis to surgery (day, range)	17.5 ± 30.5 (1–280)
Elective surgery	124 (60.8%)
Emergency surgery	80 (39.2%)
**Modalities of surgery**	
Laparotomy	49 (24.0%)
Minimal invasive surgery (laparoscopy/Robot)	155 (76.0%)
**Types of surgery**	
Cystectomy	163 (79.9%)
Salpingectomy	15 (7.4%)
Oophorectomy or salpingo-oophorectomy	23 (11.3%)
Cytoreductive surgery	3 (1.5%)
**Histopathologic results**	
Non-neoplastic tumor	72 (35.3%)
Corpus luteal cyst	24
Endometriosis	24
Simple cyst	9
Paratubal cyst	9
Hemorrhagic cyst	5
Paraovarian cyst	1
Benign neoplastic tumor	110 (53.9%)
Mature cystic teratoma	90
Serous cystadenoma	10
Mucinous cystadenoma	8
Fibroma	2
Malignant tumor	16 (7.8%)
Borderline malignancy	7
Epithelial ovarian malignancy	0
Non-epithelial ovarian malignancy	9
Ectopic pregnancy	3 (1.5%)
Tubo-ovarian abscess	3 (1.5%)

The distribution of histopathological findings of adnexal masses according to age is shown in Table 4. Mature cystic teratomas were the most common in all age groups. The incidence of endometriosis, ectopic pregnancy, and tubo-ovarian abscesses increased with age. In contrast, the incidence of malignant tumors was significantly higher in patients aged < 17 years than in those aged 18–20 years (14.5% vs. 10.7%, *P* < 0.006). All ovarian malignancies, except borderline tumors, were pathologically diagnosed as non-epithelial malignancies. The diagnostic concordance rates according to the ultrasound findings were benign neoplasms (81.8%), non-neoplastic tumors (91.1%), malignant tumors (59.1%), ectopic pregnancy (100%), and tubo-ovarian abscess (100%) (Table 5).

**Table 4:** Histopathologic diagnosis of adnexal mass according to the age.

Category	~ 14 years (n = 32)	15–17 years (n = 51)	18–20 years (n = 121)
**Non-neoplastic tumor**	10 (34.4%)	15 (29.4%)	47 (38.0%)
Endometriosis	0	3	21
Simple cyst	2	0	7
Hemorrhagic cyst	4	0	3
Corpus luteal cyst	1	8	11
Paratubal cyst	3	4	4
Paraovarian cyst	0	0	1
**Benign neoplastic tumor**	18 (53.1%)	27 (52.9%)	65 (53.7%)
Mature cystic teratoma	13	23	54
Serous cystadenoma	3	1	6
Mucinous cystadenoma	2	2	4
Fibroma	0	1	1
**Malignant tumor**	4 (12.5%)	8 (15.7%)	4 (5.8%)
Borderline	2	2	3
Epithelial	0	0	0
Non-epithelial	2	6	1
Ectopic pregnancy	0	0	3 (2.5%)
Tubo-ovarian abscess	0	1 (2.0%)	2 (1.7%)

**Table 5:** Diagnostic agreement between ultrasonography and histopathology for adnexal mass.

Categories	Sonographic diagnosis				
	Benign neoplastic tumor	Non-neoplastic tumor	Malignant tumor	Ectopic pregnancy	Tubo-ovarian abscess
Agreement	99 (81.8%)	51 (91.1%)	13 (59.1%)	2 (100%)	3 (100%)
Disagreement	22 (18.2%)	5 (8.9%)	9 (40.9%)	0	0
Histopathologic diagnosis for discrepancy cases	Endometriosis (4), Simple cyst (2), Hemorrhagic cyst (1), Corpus luteal cyst (8), Paratubal cyst (4), Borderline tumor (2), Yolk Sac tumor (1)	Mature cystic teratoma (1), Tubo-ovarian abscess (1), Serous cystadenoma (1), Fibroma (1), Ectopic pregnancy (1)	Mature cystic teratoma (4), Mucinous cystadenoma (2), Endometriosis (1), Cellular fibroma (1)	–	–

## Discussion

Adnexal masses are uncommon in children and adolescents, and they can represent a wide range of pathologies, from non-neoplastic to benign neoplasms and malignant tumors. In this study, 89.2% of the adnexal masses were benign and non-neoplastic, and only 7.8% of adnexal masses were malignant. Furthermore, mature teratomas are the most common benign ovarian tumors, consistent with the findings of recent studies^4,5,8^. Adnexal masses related to ectopic pregnancies or pelvic inflammatory disease are common in late adolescence (18–20 years). This finding seems to be associated with an increase in sexual activity with increasing age. Older teens are more likely to visit the emergency department for pelvic inflammatory disease than younger teens^9^.

The rate of malignant tumors in children and adolescents varies from 4 to 22%^10^. Germ cell tumors are the most common malignant ovarian tumors in children and adolescents. In this study, malignant tumors were found in 16 patients (seven patients with borderline tumors and nine patients with non-epithelial malignant tumors), the most common non-epithelial malignant tumor was germ cell tumor (five cases), followed by choriocarcinoma (two cases), sex cord-stromal tumor (one case), and desmoplastic small round cell tumor (one case). Patients aged < 17 years were more likely to develop non-epithelial malignancies than those aged > 18 years (*P* < 0.006).

An accurate preoperative diagnosis is challenging since the symptoms of adnexal masses are diverse and nonspecific^11^. Abdominal pain was the most common cause of surgery in the present study. It is often confused with adnexal torsion or appendicitis. Abdominal palpation and bimanual recto-abdominal or vaginal examinations in sexually active patients are required for an accurate diagnosis.

Surgical treatment of adnexal masses in children and adolescents is controversial. Removal of the suspected mass while preservation of fertility is a critical issue at this age^12,13^. In the present study, 33% of patients underwent surgery, even though they had a preoperative diagnosis of non-neoplastic tumors on ultrasonography. These patients had a mean adnexal mass size of 6.3 cm, and could not be differentiated from adnexal torsion due to severe abdominal pain. However, 10.9% of patients diagnosed with neoplastic tumors did not undergo surgery because of incidental findings and small-sized adnexal masses.

Various modalities can be used to diagnose adnexal masses and determine treatments. Most masses are detected using ultrasonography, which is the first-line imaging test^14^. Because of its high accessibility, relatively low risk, cost-effectiveness, and diagnostic accuracy, ultrasonography is a useful diagnostic tool for differentiating adnexal masses^15^. Ultrasound findings are good indicators of whether the patient should be operated or managed conservatively^16^. Furthermore, ultrasonography allows continuous imaging follow-up for relatively small ovarian masses without surgical treatment^17^. The results of this study demonstrated that the size of non-neoplastic tumors was smaller than that of neoplastic masses. Patients who underwent surgery had large tumors and showed neoplastic features on ultrasonography compared to those who were observed without surgery (*P* < 0.0001). Reassuringly, the ultrasonographic diagnosis was consistent with the histopathological diagnosis (k = 0.722, *P* < 0.0001). A previous study has shown that 90% of simple cysts measuring 5–7 cm on ultrasonography decreased in size or resolved on follow-up^18^.

In addition to ultrasonography, CT or MRI can be helpful in the diagnosis of malignant ovarian tumors with high accuracy. Additional information, such as the nature of the adnexal mass and metastatic involvement of the pelvic and para-aortic lymph nodes can be determined with CT or MRI^19,20^. In malignant ovarian tumors, the levels of serum tumor markers (AFP, β-hCG, CA-125, CA-19-9, and CEA) tended to rise^21^. However, in this study, 44% of patients with malignant tumors had normal levels of serum tumor markers. Approximately 50% of malignant tumors present with elevated tumor marker values^22^. Therefore, normal serum tumor marker levels cannot exclude a malignancy.

Generally, if a malignant tumor is not suspected, ovarian-sparing surgery is the standard treatment for benign ovarian tumors^23,24^. Surgery for benign ovarian tumors is conservative, and ovarian cystectomy or simple excision is usually performed^25,26^. Ovarian-sparing surgery has successful clinical outcomes, with low rates of recurrence and repeated surgery^7,26^. Approximately 87% of the patients in this study underwent ovarian-sparing surgery. Most patients underwent MIS (laparoscopy or robotic surgery), and none of the patients required a laparotomy. MIS has been widely used in many surgeries, including those involving the female genital tract^27^. MIS is associated with greater cost-effectiveness, less pain, shorter hospitalization, reduced recovery time, lower incidence of surgical site infection, less bleeding, more satisfaction with scars, and fewer postoperative complications than laparotomy surgery^28,29^. Because of these advantages, MIS usage has increased over time (2005–2018) to treat young women with adnexal masses in our institution. However, the choice between laparotomy and MIS, especially if a malignant tumor is suspected, is controversial.

In conclusion, abdominal pain is the most common reason for hospital visits and surgery in adolescents and young women with adnexal masses. The ultrasonographic diagnosis was consistent with histopathological diagnosis. In recent years, ovarian-sparing surgery with laparoscopy or robotic surgery has been increasingly used for the treatment of young patients with adnexal masses. Long-term follow-up is needed to fully assess the effects of ovarian-sparing surgery on future fertility and ovarian function in this population.

## Methods

This retrospective study included young women aged < 20 years, diagnosed with adnexal masses between January 2005 and December 2018 at the Konkuk University Medical Center. Patients with secondary ovarian malignancies or a history of malignancy were excluded. After obtaining institutional review board approval (No. KUMC 2020-04-055), we reviewed the medical charts of the patients, including clinical characteristics and surgical and histopathological reports. Data on presenting symptoms, age at diagnosis, tumor size on ultrasonography, surgical procedures, and serum tumor markers were extracted. According to the surgical reports, the type of surgery was defined as (1) cystectomy, (2) salpingectomy, (3) oophorectomy or salpingo-oophorectomy; and (4) cytoreductive surgery, including unilateral salpingo-oophorectomy, pelvic lymphadenectomy, omentectomy, and peritoneal washing.

### Statistical analysis

Categorical variables are presented as numbers and percentages, and continuous variables are presented as means with standard deviations. To assess differences between groups, we used the *t* test and chi-square test for continuous and categorical variables, respectively. Cohen's kappa statistic was used to evaluate the diagnostic agreement between the ultrasonography and histopathological findings. Linear trend estimation was used to make statements about tendencies in surgery from 2005 to 2018. The R^2^ statistic shows how significantly the slope of the fitted line differs from zero. Statistical analyses were performed using IBM SPSS (version 21.0; SPSS Inc., Chicago, IL, USA). Statistical significance was set at *P* < 0.05.

### Ethical approval

The study was approved by the Institutional Review Board of Konkuk University Medical Center (No. KUMC 2020-04-055). All procedures performed in this study were in accordance with the ethical standards of the institution and with the 1964 Helsinki Declaration and its later amendments. Informed consent was obtained from all participants from a parent and/or legal guardian, as the patients involved in the study were below 18 years of age.

## Data Availability

The datasets used and analyzed during the current study are provided by the corresponding author upon reasonable request.
